# Fission yeast essential nuclear pore protein Nup211 regulates the expression of genes involved in cytokinesis

**DOI:** 10.1371/journal.pone.0312095

**Published:** 2024-12-12

**Authors:** Domenick Kamel, Ayisha Sookdeo, Ayana Ikenouchi, Hualin Zhong

**Affiliations:** 1 Department of Biological Sciences, Hunter College, The City University of New York, New York, NY, United States of America; 2 The Graduate Center, The City University of New York, New York, NY, United States of America; 3 Department of Science and Mathematics, Guttman Community College, The City University of New York, New York, NY, United States of America; University of Cambridge, UNITED KINGDOM OF GREAT BRITAIN AND NORTHERN IRELAND

## Abstract

Nuclear pore proteins control nucleocytoplasmic transport; however, certain nucleoporins play regulatory roles in activities such as transcription and chromatin organization. The fission yeast basket nucleoporin Nup211 is implicated in mRNA export and is essential for cell viability. Nup211 preferentially associates with heterochromatin, however, it is unclear whether it plays a role in regulating transcription. To better understand its functions, we constructed a *nup211* “shut-off” strain and observed that Nup211 depletion led to severe defects in cell cycle progression, including septation and cytokinesis. Using RNA-Seq and RT-qPCR, we revealed that loss of Nup211 significantly altered the mRNA levels of a set of genes crucial for cell division. Using domain analysis and CRISPR/cas9 technology, we determined that the first 655 residues of Nup211 are sufficient for viability. This truncated protein was detected at the nuclear periphery. Furthermore, exogenous expression of this domain in *nup211* shut-off cells effectively restored both cell morphology and transcript abundance for some selected genes. Our findings unveil a novel role for Nup211 in regulating gene expression.

## Introduction

Nuclear pore complexes (NPCs) mediate the bidirectional exchange of materials between the nucleus and cytoplasm. Each NPC is composed of about thirty different proteins, which are collectively called nucleoporins (or Nups) [1–3]. Nups assemble into several sub-structures including the cytoplasmic filaments, central core, and nuclear filaments. Both the cytoplasmic and nuclear filaments associate with the core embedded in the nuclear envelope. While the cytoplasmic filaments extend outward into the cytoplasm, the nuclear filaments extend into the nucleoplasm and merge at a ring to form a basket-like structure. NPCs exhibit eight-fold rotational symmetry about their central axis and their central cores have 2-fold mirror symmetry across the midplane of the nuclear envelope. However, the cytoplasmic and nuclear filaments make the NPCs vertically asymmetric. Due to these features, Nups are usually present in multiples of eight. In addition to their structural roles, many Nups actively participate in nucleocytoplasmic transport as well as other cellular functions like transcription, DNA repair, and cell cycle regulation [4–7]. In this work, we investigated the roles of Nup211, a nucleoporin associated with the nuclear basket in the fission yeast *Schizosaccharomyces pombe*.

The Nup211 protein consists of 1837 amino acids (calculated MW of ~211 kDa) and is essential for cell viability [8]. Nup211 is localized at the periphery in the nucleus and relocates to the nucleolar ring upon heat shock [9–11]. Previous studies showed that Nup211 plays a role in mRNA export as its overexpression or down-regulation results in the nuclear accumulation of polyA-RNA [12]. In addition, Nup211 has been implicated in mRNA quality control, as overexpressing Nup211 inhibits the nuclear export of intron-containing RNA [13]. The underlying mechanisms by which Nup211 regulates these processes remain unknown.

Nup211 is evolutionarily conserved from yeast to humans. The mammalian ortholog TPR (translocated promoter region) is a 267 kDa filamentous protein, also located at the nuclear basket and extends into the nuclear interior [14–16]. Recent studies identified two subpopulations of TPR which are located at the nuclear basket through binding to different nuclear pore proteins [17–19]. The nucleoporin Alm1 (abnormal long morphology) is considered to be another TPR ortholog in *S*. *pombe* [20]. Alm1 is important for proper chromosome segregation, however, it is not essential for cell viability [21]. There are also two TPR orthologs in *Saccharomyces cerevisiae*, called myosin-like proteins Mlp1 and Mlp2. Interestingly, neither is essential for viability. Even though nuclear import and cell fitness are affected, the strain with both *Mlp1* and *Mlp2* deleted is viable [22]. Megator is considered the only TPR ortholog in *Drosophila melanogaster* and is essential for viability [23].

The amino-terminal two-thirds of TPR mostly consists of coiled-coil motifs, which have been shown to be important for dimerization [24]. Together with other nuclear proteins, TPR and its orthologs (TPR proteins) can form higher-order structures that serve as anchoring sites for proteins involved in various nuclear activities [for reviews, see [25–27]]. For example, the TPR proteins interact with mitotic spindle checkpoint proteins Mad1 and Mad2, recruiting them to the NPC during interphase [28–30]. Mammalian TPR remains associated with Mad1/Mad2 and is located at the kinetochore during mitosis [29]. TPR also directly interacts with extracellular signal-regulated kinase 2 (ERK2) and anchors it at nuclear pores. Interestingly, ERK2 phosphorylates TPR, enhancing their interaction [31, 32]. The budding yeast Mlp proteins interact with components of the Spt-Ada-Gcn5-acetyltransferase (SAGA) complex and recruit transcriptionally active genes to the nuclear periphery [33]. The Mlps are also required for properly positioning the SUMO protease Ulp1 near the NPCs, which is important for regulating the SUMO status of transcription factors [34, 35]. In fission yeast, Mad1/Mad2 and Ulp1 are localized to the nuclear periphery during interphase [36, 37]; however, it is unknown whether Nup211 is important for the proper localization of these proteins.

Nup211 orthologs can regulate gene expression at multiple levels such as chromatin organization, transcription, and mRNA export [12, 38–42]. For example, TPR establishes the borders of heterochromatin around NPCs [43, 44], while Mlp2 plays a role in anchoring telomeres at the nuclear periphery [45]. Interestingly, ChIP-Seq revealed that Nup211 preferentially associates with heterochromatin [46]. However, it is not clear whether Nup211 controls gene expression at the chromatin level. To better understand its functions, we constructed a *nup211* depletion strain to study how loss of the protein affects cellular activities. Here, we report that the essential function of Nup211 is located in the first 655 residues. Down-regulation of Nup211 led to severe defects in the cell cycle regulation, including septation and cytokinesis. Ectopically expressing full length Nup211 or Nup211_1-655_ showed a rescue effect. RT-qPCR results confirmed that Nup211 controls the expression of a set of genes involved in cytokinesis, suggesting a novel role for Nup211 in regulating gene expression.

## Results

### Generating and characterizing a conditional *nup211* mutant strain

**Fig 1:**
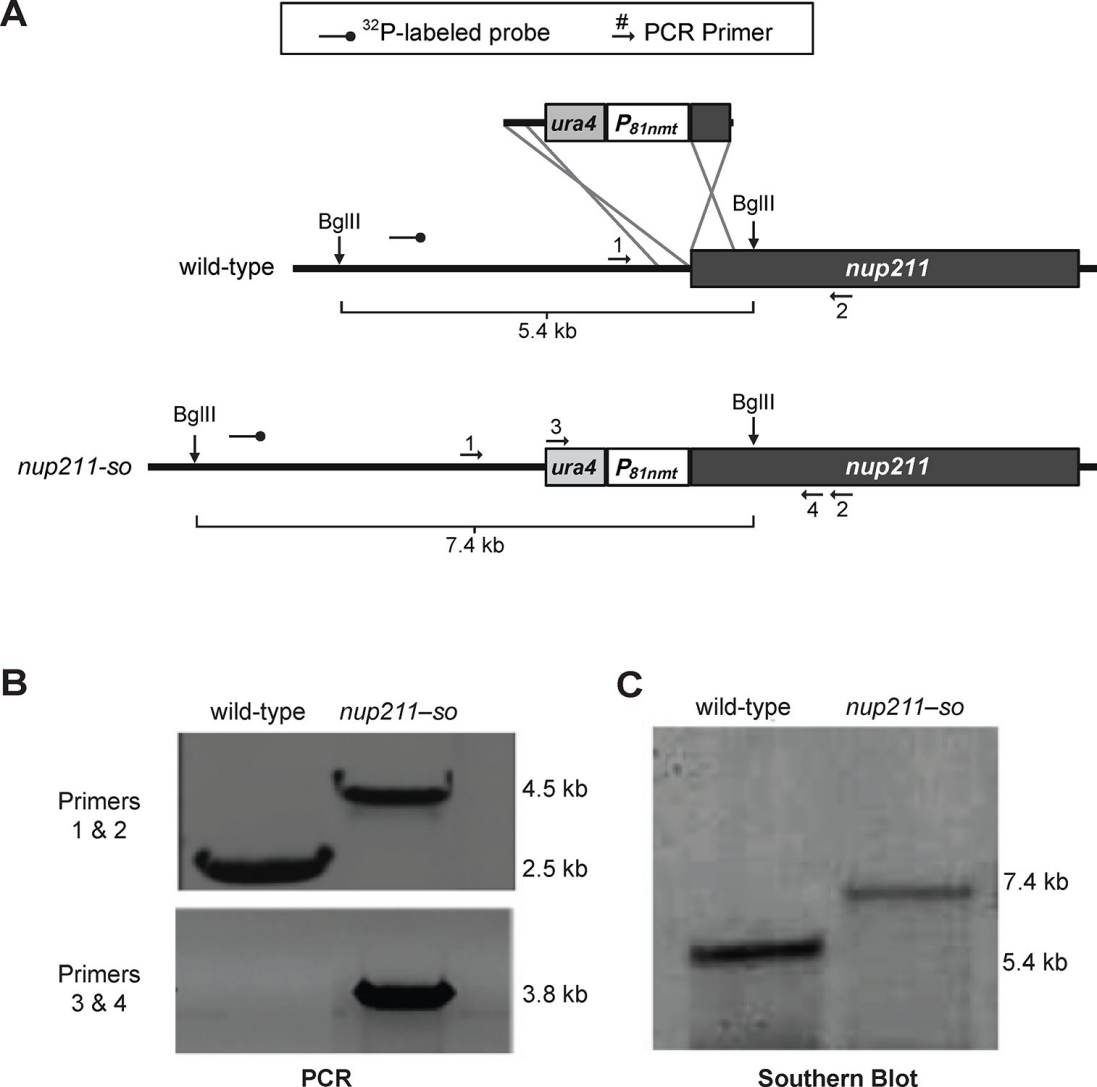
Verification of the *nup211* shut-off (*nup211-so*) strain. (A) Schematic of the *nup211* loci of wild-type (*nup211*^*+*^) and *nup211-so* strains. Primer 1 is complementary to a region upstream of the *nup211* open reading frame (ORF), primers 2 and 4 are complementary to the *nup211* ORF, and primer 3 is complementary to the *ura4*^*+*^ gene. (B) PCR and (C) Southern blot analysis results confirmed the integration of *ura4*^*+*^ and P_81nmt_ upstream of the *nup211* locus.

Previous studies have revealed that *nup211* is an essential gene [8]. To elucidate the functions of Nup211, we therefore generated a conditional mutant strain named *nup211-so* (*nup211*-shut off), in which the thiamine regulatory *nmt1* promoter P81nmt was inserted upstream of the *nup211* coding sequence. To construct this strain, we used PCR to synthesize a DNA fragment containing P81nmt and the *ura4*^*+*^ gene flanked by 400 bp sequences homologous to parts of the endogenous *nup211* promoter and ORF (Fig 1A). The purified DNA fragment was transformed into strain 558 (S1 Table) and was integrated into the *nup211* locus via homologous recombination. We first examined the integration event by PCR (Fig 1B). Using primers 1 & 2, a 2.5 Kb fragment was amplified from wild-type genomic DNA while a 4.5 Kb fragment was amplified from *nup211-so* genomic DNA (Fig 1A and S2 Table). When primers 3 & 4 were used, a 3.8 Kb fragment was generated only when *nup211-so* genomic DNA was used as template (Fig 1B, lower panel). The PCR results confirmed that P81nmt is located upstream of the *nup211* coding sequence. We further validated integration by Southern blot using a radioactively labeled probe complementary to the endogenous *nup211* promoter (Fig 1C and S2 Table). A 5.4 Kb fragment and a 7.4 Kb fragment were detected following digestion of wild-type and *nup211-so* genomic DNA, respectively (Fig 1). These results confirmed the integration of P81nmt and *ura4*^*+*^ at the *nup211* locus.

**Fig 2:**
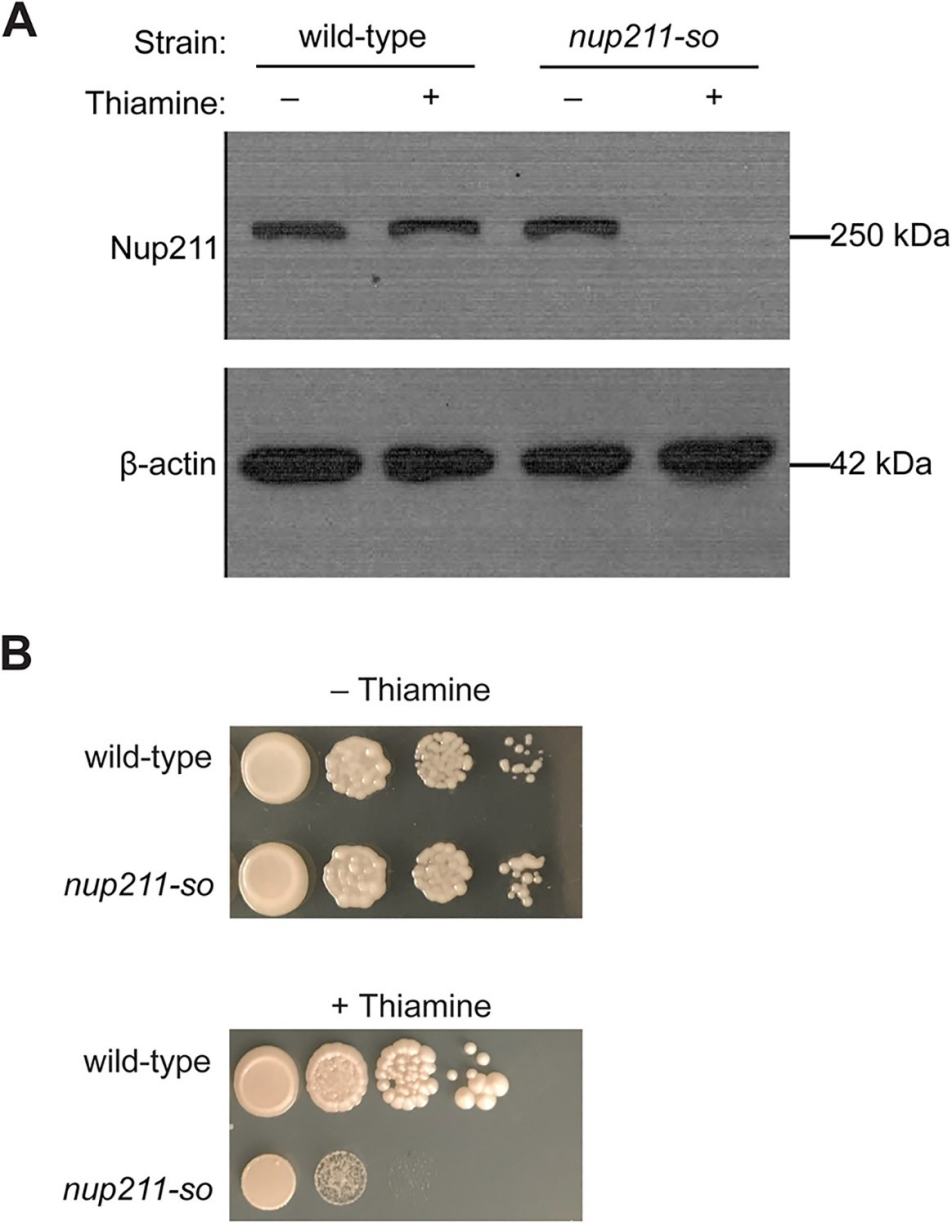
Characterization of the nup211-so strain. (A) Comparison of Nup211 protein levels in wild-type and *nup211-so* cells grown in medium with or without thiamine by immunoblotting with anti-Nup211 antibodies. β-actin was used as a protein loading control. (B) Growth rate comparison between wild-type and *nup211-so* cells grown on medium with or without thiamine. Spot assays started with 5x10^4^ cells, followed by tenfold series dilution.

To determine whether *nup211* expression is controlled by P81nmt in the *nup211-so* strain, we grew cells in medium containing 10 μg/mL thiamine and examined Nup211 protein levels. Western blotting showed that Nup211 protein level was significantly reduced in *nup211* shut-off cells compared to non-shut-off and wild type controls (Fig 2A). We also performed spot assays to analyze the growth of *nup211-so* cells. In the presence of thiamine, *nup211-so* cells showed a reduced viability of approximately 1000-fold when compared with non-shut-off cells. In addition, *nup211-so* colonies in the presence of thiamine were small, showing a severe growth defect (Fig 2B).

### Shutting-off *nup211* resulted in defects in cell morphology and cytokinesis

**Fig 3:**
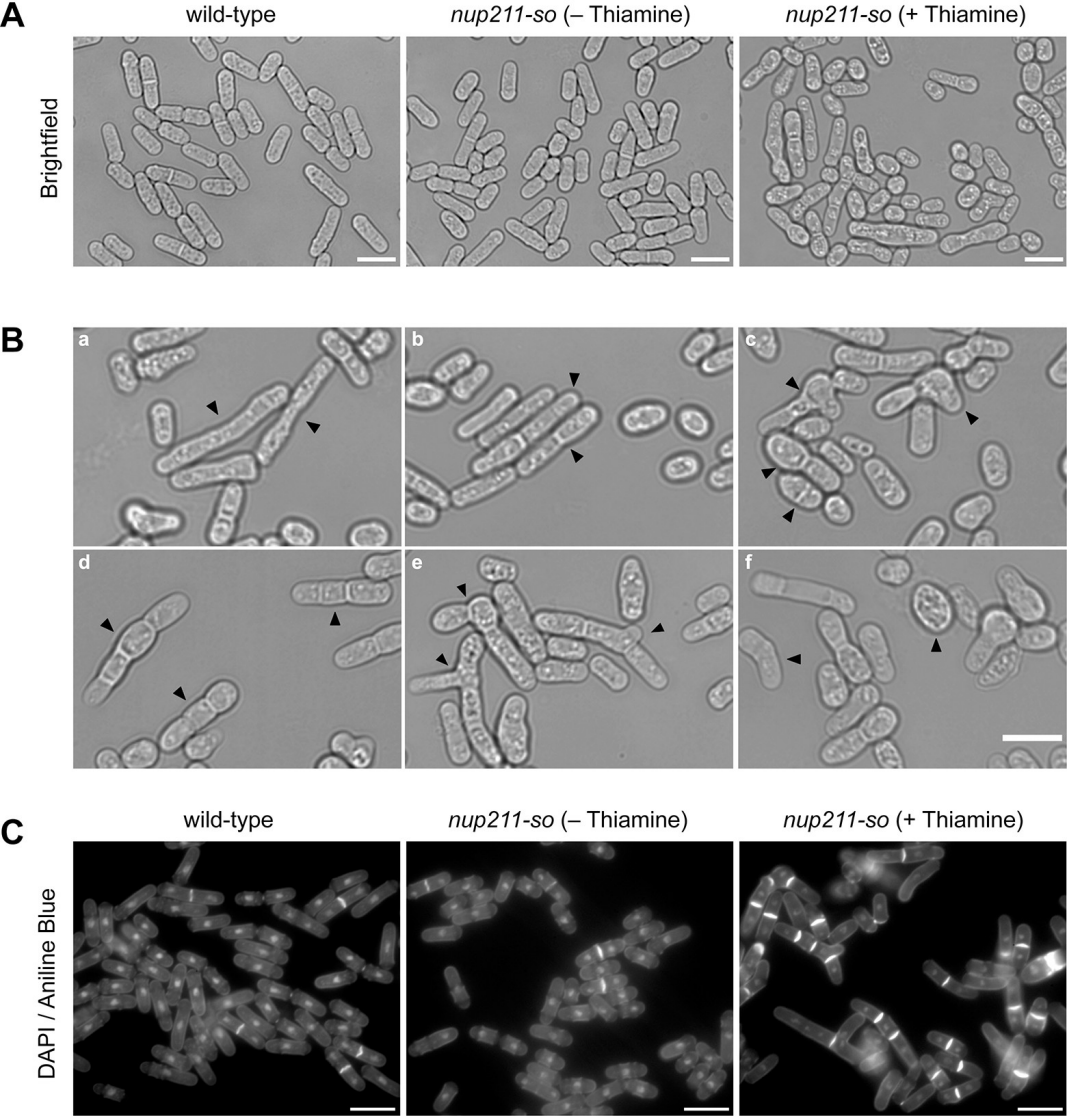
Down-regulation of Nup211 led to severe defects in cell morphology and cytokinesis. (A) Bright-field images of wild-type and *nup211-so* cells grown in medium with or without thiamine. (B) Representative images of *nup211-so* cells grown in medium containing thiamine. Cells exhibit various morphological defects: elongated without septa (a), thick septa (b), asymmetric septa or bulged shape (c), multiseptated (d), branched morphology (e), and curved or swollen shape (f). Septa could also be observed in shorter cells (c). Arrowheads point to cells with specific defects. (C) Wild-type and *nup211-so* cells stained with DAPI (nuclei) and aniline blue (septa). For all panels, *nup211-so* cells were grown in medium containing thiamine for 48 hours prior to imaging. Bars: 10 μm.

To better understand why Nup211 down-regulation significantly impairs cell viability (Fig 2B), we examined *nup211-so* cells by microscopy. Shutting off *nup211* expression led to a wide range of morphological defects (Fig 3A). Some cells appeared abnormally round, curved, bulged, or branched, indicating that Nup211 plays a role in maintaining proper cell shape (Fig 3B). Interestingly, *nup211* shut-off cells also showed severe defects in septation and cytokinesis. These defects varied widely: some cells failed to develop a septum during division (Fig 3B-a), while others developed thicker (Fig 3B-b), misplaced (Fig 3B-c), or multiple septa (Fig 3B-d). Furthermore, septa were sometimes seen in shorter cells (Fig 3B-c), while other phenotypes like bulging (Fig 3B-c, 3B-e), branching (Fig 3B-e), curving, and swelling (Fig 3B-f) were also observed.

To better analyze septation and nuclear division in *nup211* shut-off cells, we stained nuclei and septa with DAPI and aniline blue, respectively. Compared with wild type cells, a higher percentage of *nup211* shut-off cells contained multiple and/or thicker septa (Fig 3C). Taken together, our data suggest that Nup211 plays roles in cytokinesis in addition to regulating cell shape.

### Domain analysis showed that the N-terminal region of Nup211 is sufficient for cell viability

**Fig 4:**
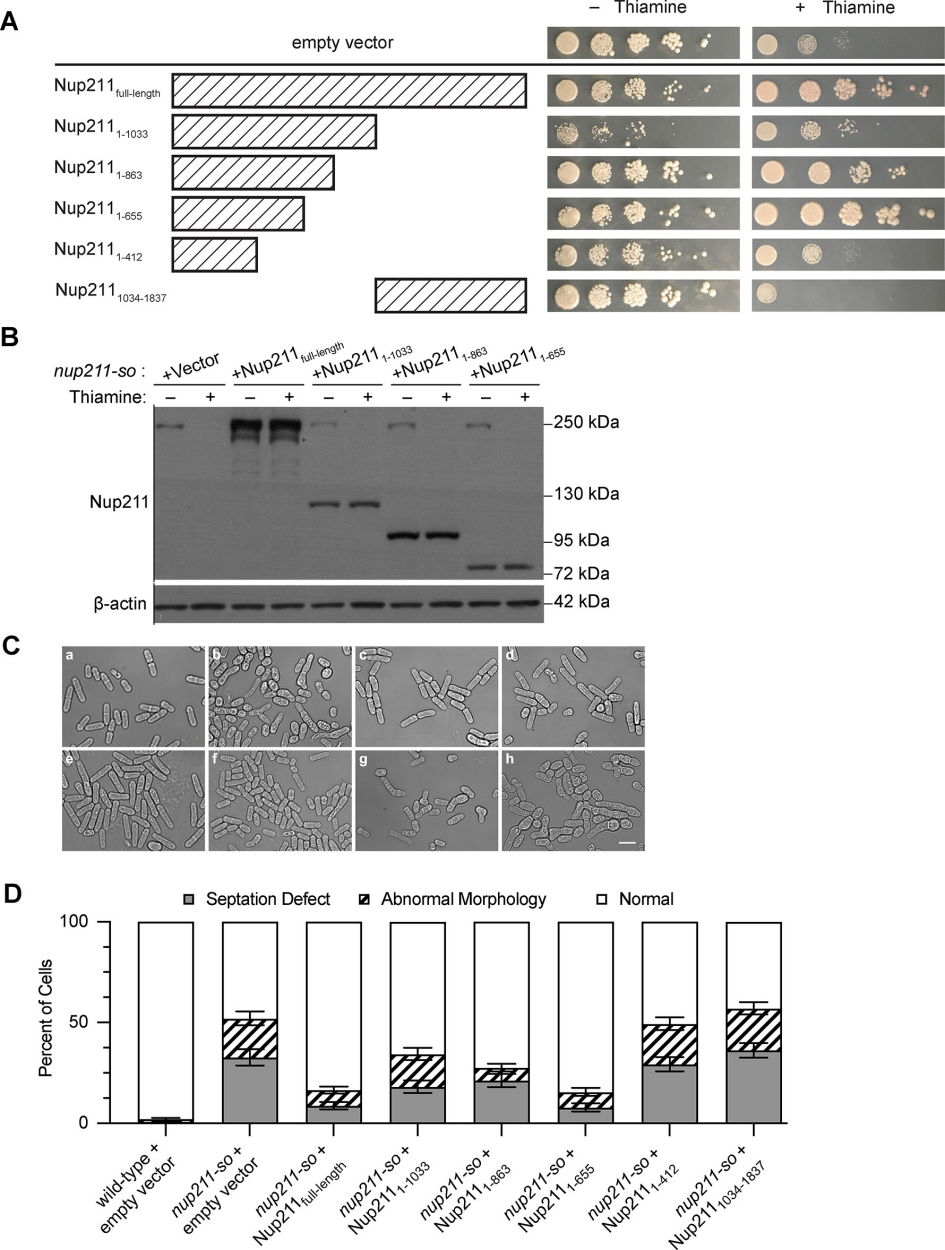
The N-terminal region of Nup211 is sufficient for cell viability. (A) Growth analysis of *nup211-so* cells transformed with constructs expressing full-length or fragments of Nup211 grown in the presence or absence of thiamine. Spot assays started with 5x10^5^ cells, followed by tenfold series dilution. (B) Examining Nup211 protein levels in the strains shown in (A) by immunoblotting with anti-Nup211 antibodies. β-actin was used as a protein loading control. (C) Representative bright-field images of (a) wild-type and (b) *nup211-so* cells transformed with empty vector. Panels (c-d) show images of *nup211-so* cells expressing full-length (c) or fragments of Nup211: (d) Nup211_1-1033_, (e) Nup211_1-863_, (f) Nup211_1-655_, (g) Nup211_1-412_, and (h) Nup211_1034-1837_. Bar: 10 μm. (D) Distribution chart showing the rescuing effects of strains with the representative images shown in (C). At least 500 cells were analyzed in each condition.

Given that *nup211* shut-off cells showed reduced viability along with severe defects in morphology and cytokinesis, we wanted to know whether exogenous expression of certain Nup211 domains could have a rescue effect. To address this question, we constructed plasmids that express different regions of Nup211 under the constitutive *adh1* promoter, transformed them into *nup211-so* cells, and determined the effects on viability through spot assays (S1 Table). Exogenous expression of Nup211, Nup211_1-863_, or Nup211_1-655_ restored cell viability; however, expression of Nup211_1-1033_ only led to a partial recovery (Fig 4A). Nup211_1-412_ was unable to rescue cell viability, while the C-terminal region Nup211_1034-1837_ further decreased the viability of *nup211* shut-off cells. Interestingly, expressing Nup211_1-1033_ in non-shut-off cells slightly reduced viability, indicating that excess Nup211_1-1033_ inhibited cell growth.

To determine if exogenous expression of Nup211 domains could also rescue the morphological defects of *nup211* shut-off cells, we analyzed shut-off cells harboring these rescue constructs by microscopy. Consistent with spot assay results, Nup211_1-863_ and Nup211_1-655_ showed a significant rescue effect, but Nup211_1-412_ did not (Fig 4C and 4D). Similarly, Nup211_1-1033_ could not rescue cell morphology as effectively as Nup211_1-863_ or Nup211_1-655,_ suggesting that residues 864–1033 have some inhibitory effect. Lastly, expression of Nup211_1034-1837_ exacerbated the morphology defects, indicating that expression of this domain is toxic to cells. Further investigation is needed to identify the functions of Nup211_864-1033_ and Nup211_1034-1837_.

**Fig 5:**
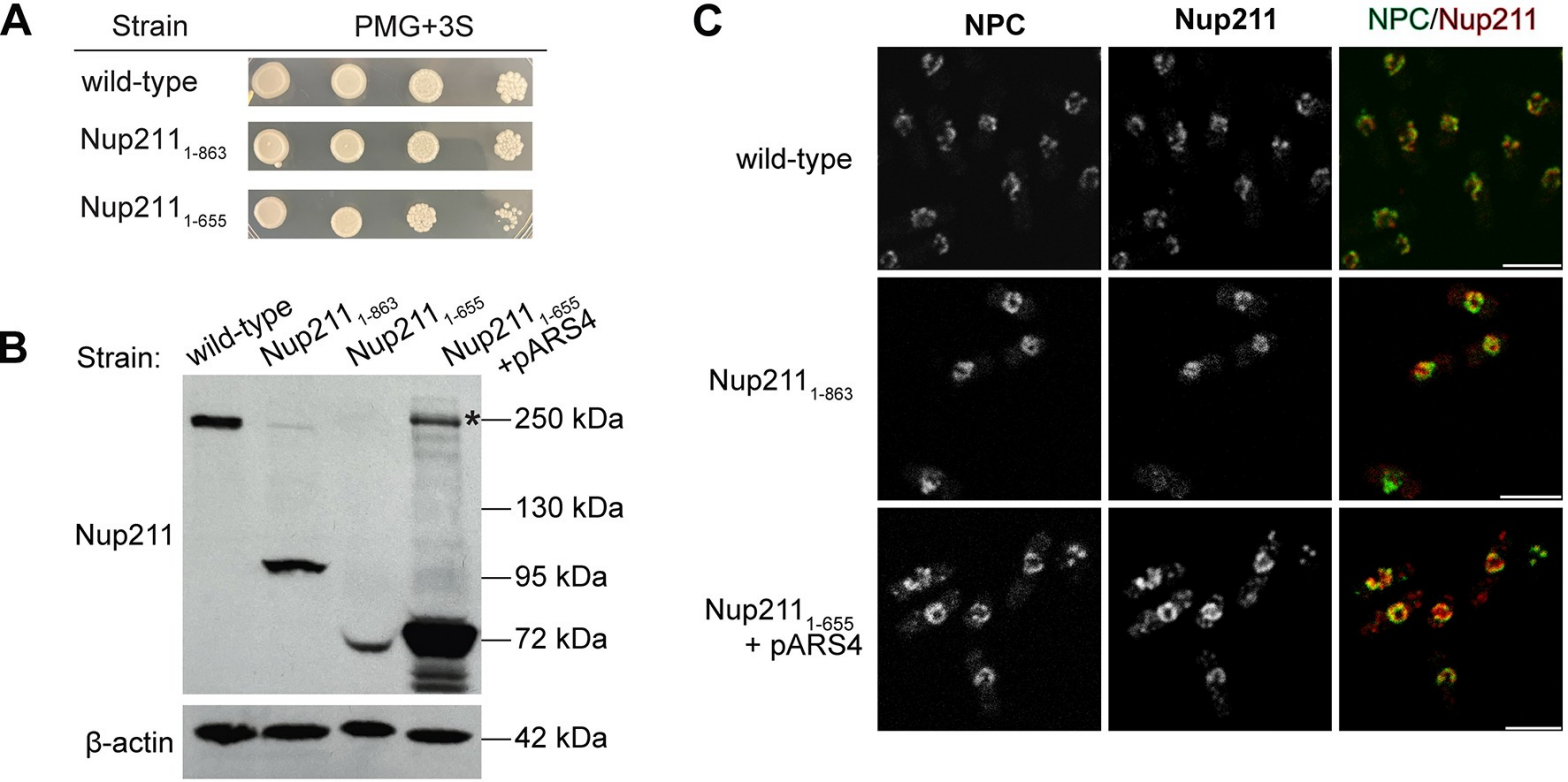
Analyses of Nup211 truncation strains confirmed that the N-terminal 655 residues are sufficient for cell viability. (A) Growth analysis of wild-type, *nup211*_*1-863*_ and *nup211*_*1-655*_ strains. Spot assays started with 1x10^5^ cells, followed by tenfold series dilution. (B) Examining Nup211 protein levels in wild-type, *nup211*_*1-863*_, *nup211*_*1-655*_, and *nup211*_*1-655*_ + pARS4 (expressing Nup211_1-655_) strains by immunoblotting with anti-Nup211 antibodies. β-actin was used as a protein loading control. * high order (possible tetramer) of Nup211_1-655_. (C) Representative confocal images of wild-type, *nup211*_*1-863*_, and *nup211*_*1-655*_ + pARS4 cells immunostained with anti-Nup211 and mAb414 antibodies. mAb414 recognizes several nucleoporins of NPCs. Bars: 5μm.

Our domain analysis using *nup211-so* strain suggests that Nup211_1-655_ is sufficient to carry out the essential function(s) of Nup211. Even though we did not detect endogenous Nup211 protein in *nup211* shut off cells (Fig 4B), we cannot exclude the possibility that residual undetected Nup211 protein contributed to the rescue effects. To address this issue, we used the CRISPR system to generate mutant strains (*nup211*_*1-863*_ and *nup211*_*1-655*_) in which the *nup211* gene is truncated at its chromosomal locus (S1 and S2 Tables) [47]. Both *nup211*_*1-863*_ and *nup211*_*1-655*_ cells were viable and grew as well as the wild-type strain on PMG plates, confirming that the N-terminal 655 residues of Nup211 are sufficient for viability (Fig 5A).

The N-terminal 655 residues of Nup211 carry out its essential functions, since *nup211*_*1-655*_ cells were viable (Fig 5A). Although we were able to detect a very low amount of Nup211_1-655_ by Western blotting (Fig 5B), we were not able to detect and localize Nup211_1-655_ in the *nup211*_*1-655*_ cells using immunofluorescence microscopy. It’s possible that Nup211_1-655_ is expressed at a low level and/or is easily degraded. As shown in Fig 4B, full-length and truncated Nup211 proteins were detected at different levels even though they were expressed from the same expression vector under the *adh1* promoter. Notably, the abundance of Nup211_1-655_ and Nup211_1-1033_ was lower than that of Nup211_1-863_, indicating that the position of protein truncation affects the protein level. To help determine the location of Nup211_1-655_ in the CRISPR-modified strain, we introduced the plasmid pARS4 to increase the expression of Nup211_1-655_ (Fig 4). As expected, the Western blotting results showed that Nup211_1-655_ protein level was significantly increased in the transformed cells (Fig 5B). Furthermore, we were able to detect Nup211_1-655_ at the nuclear periphery, similar to the localization of full-length Nup211 (Fig 5C).

### Down-regulation of Nup211 affected mRNA levels of genes involved in cytokinesis

**Fig 6:**
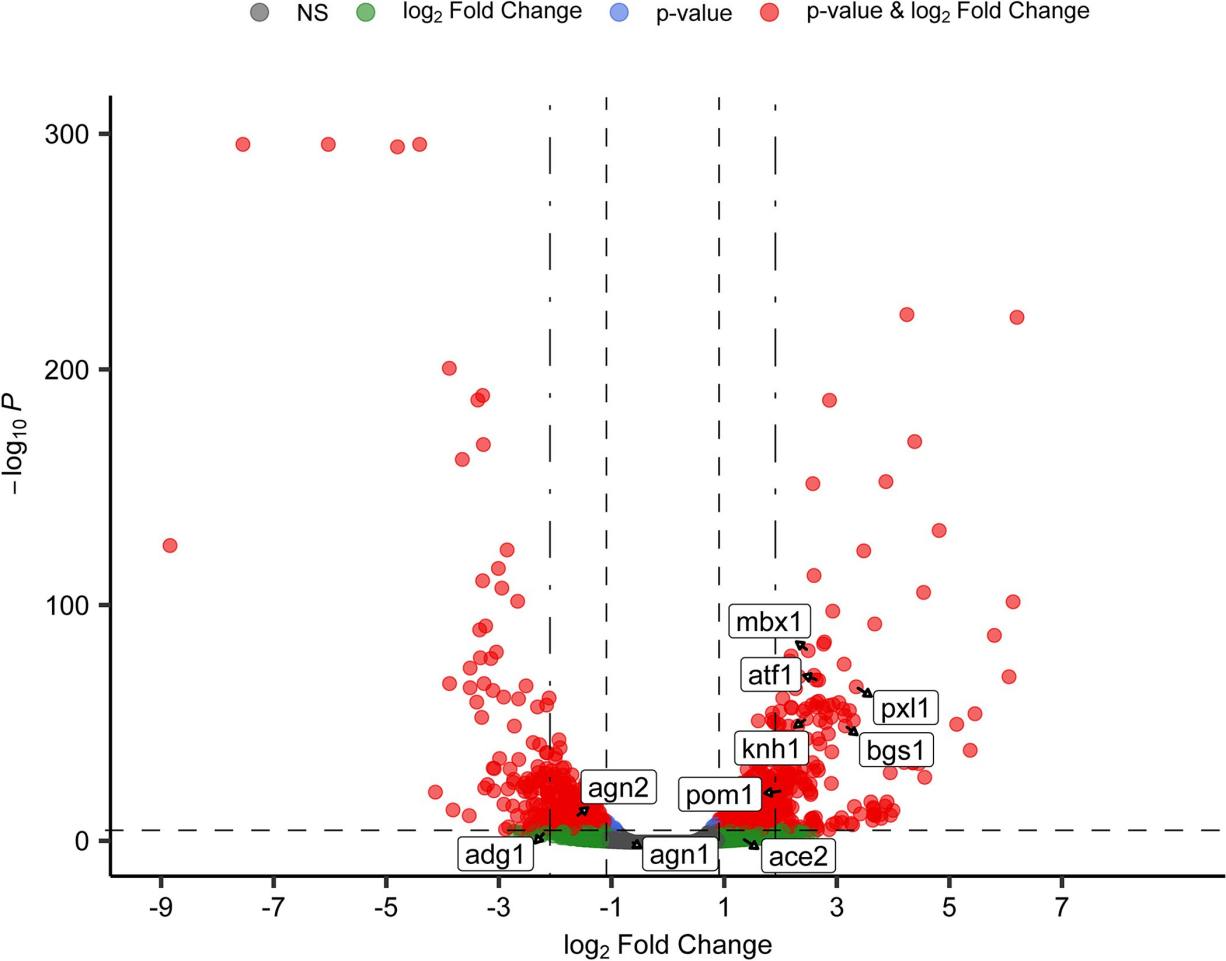
Transcript abundance was significantly altered upon down-regulation of *nup211*.

Our previous results confirmed that Nup211 is essential for cell viability and revealed that the essential region of Nup211 is located within the N-terminal 655 residues. However, the mechanism by which Nup211 impacts cell viability remains unknown. It has been reported that Nup211 and its orthologs play roles in mRNA export [38, 42, 48–50]. Several orthologs of Nup211 have also been implicated in gene regulation at the chromatin level [35, 44, 51–53]. To determine whether Nup211 plays a role in transcriptional regulation, we performed RNA-Seq using the *nup211-so* strain. Our RNA-seq results revealed that the mRNA levels of many genes were significantly altered upon the down-regulation of *nup211* (Fig 6 and S3 Table). Among them are a number of genes involved in cell cycle regulation, including cytokinesis, which is consistent with our microscopy results (Fig 3). Thus, we selected several representative genes and further analyzed their mRNA levels via RT-qPCR.

The volcano plot shows changes in transcript abundance between *nup211-so* cells cultured in thiamine-supplemented media and those grown without thiamine. Each point in the plot represents an individual gene. The x-axis displays the log_2_ normalized fold change in expression, while the y-axis shows the -log_10_-transformed p-values and reflects statistical significance. The horizontal line represents the default p-value cutoff used by the *enhancedVolcano* R program (10^−6^) and the two vertical lines indicate the fold-change cutoffs of two-fold and four-fold. Transcripts meeting the criteria for statistical significance (p-value less than 10^−6^) and differential expression (fold change greater than two-fold) are highlighted in red, while those that are significant only by p-value or fold change alone are indicated in blue or green, respectively. Non-significantly affected transcripts are shown in gray. Transcripts labeled on the plot (*atf1*, *mbx1*, *bgs1*, *knh1*, *pxl1*, *pom1*, *ace2*, *adg1*, *agn1*, and *agn2*) were analyzed further by RT-qPCR.

**Fig 7:**
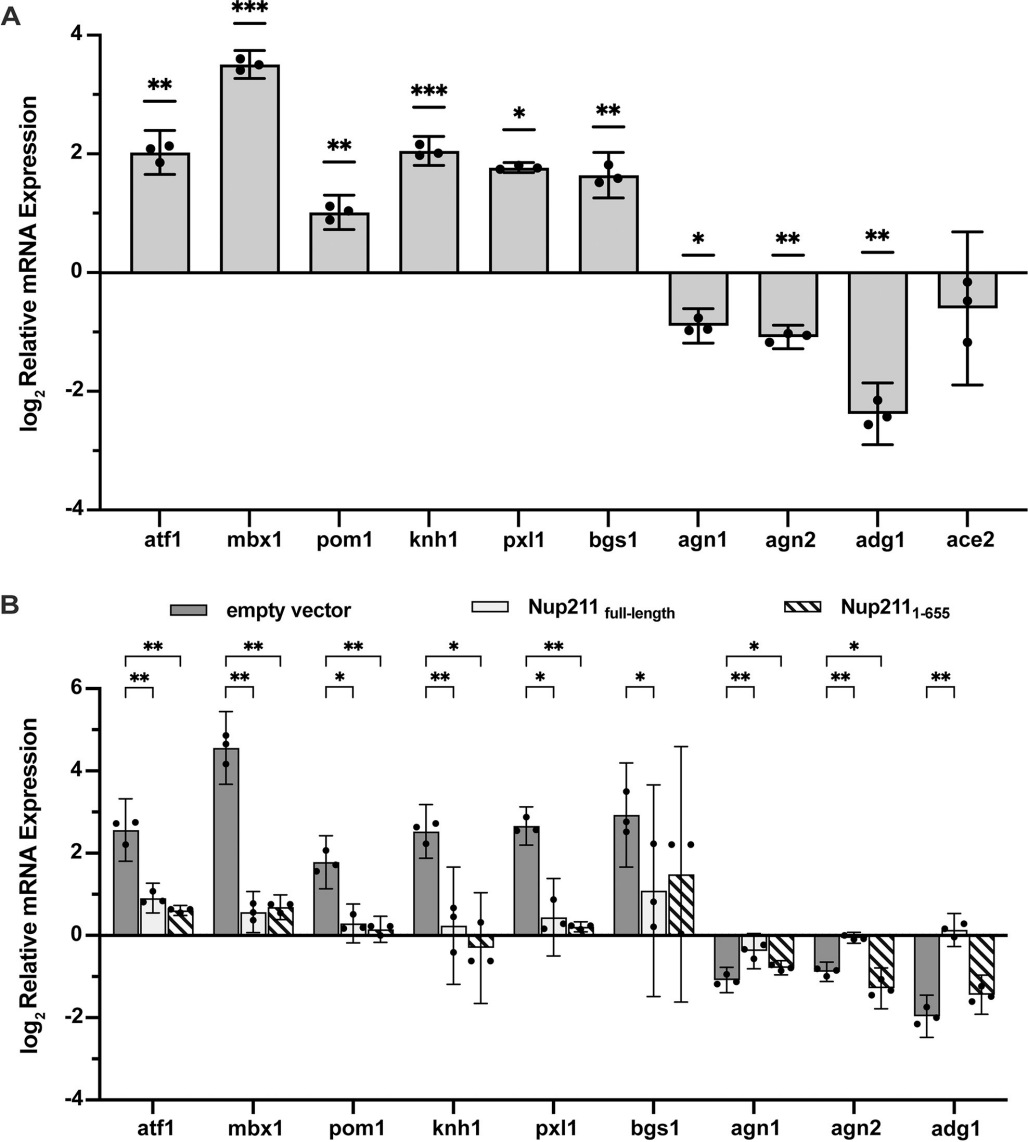
Nup211 regulates the expression of several genes involved in cytokinesis. (A) Analyzing the mRNA levels of representative genes (*atf1*, *mbx1*, *pom1*, *knh1*, *pxl1*, *bgs1*, *agn1*, *agn2*, *adg1* and *ace2*) via RT-qPCR. (B) Analyzing the mRNA levels of representative genes in *nup211-so* cells exogenously expressing full-length Nup211 or Nup211_1-655_ via RT-qPCR. For both (A) and (B), each transcript was analyzed in triplicate for all experimental conditions. Changes in relative transcript levels were calculated using the ΔΔC_q_ method, with *act1* serving as the endogenous control. Error bars represent the 95% confidence interval. Individual points represent the independent data values from the biological replicates. (* p < 0.05, ** p <0.01, *** p <0.001).

The genes we analyzed are transcription factors *atf1* and *mbx1*, DYRK family cell polarity protein kinase *pom1*, conserved fungal cell wall protein *knh1*, paxillin-like protein *pxl1*, linear 1,3-beta-glucan synthase catalytic subunit *bgs1*, endo-1,3-alpha-glucosidases *agn1* and *agn2*, and *ace2* dependent gene *adg1*. After shutting off *nup211* expression, the mRNA levels of *atf1* (4.1-fold), *mbx1* (11.3-fold), *pom1* (2-fold), *knh1* (4.1-fold), *pxl1* (3.4-fold), and *bgs1* (3.1-fold) were significantly increased while the mRNA levels of *agn1* (0.53-fold), *agn2* (0.47-fold) and *adg1* (0.19-fold) were significantly decreased (Fig 7A and S4 Table). Since *ace2* is a transcriptional activator for several genes controlling cytokinesis, we also examined *ace2* even though it wasn’t significantly affected in our RNA-Seq data [54]. We confirmed that *ace2* mRNA level was not significantly changed by shutting off Nup211 expression. Consistent with the RNA-Seq data, our RT-qPCR results indicate that Nup211 regulates mRNA levels of genes involved in cytokinesis.

### Ectopically expressing Nup211 rescued the expression of several genes involved in cytokinesis

Since the N-terminal domain of Nup211 (Nup211_1-655_) was sufficient to restore cell viability when endogenous *nup211* expression was shut off (Fig 4A), we wondered if ectopically expressing this region could restore the mRNA levels of some genes we previously examined. To address this question, mRNAs from *nup211-so* cells harboring plasmids expressing either Nup211 or Nup211_1-655_ were isolated to serve as templates for RT-qPCR. Compared with the vector control, full-length Nup211 showed significant rescue effects for all the target genes (Fig 7B). Interestingly, expressing Nup211_1-655_ was able to fully or partially rescue the expression of most affected genes (*atf1*, *mbx1*, *pom1*, *knh1*, *pxl1 and agn1*), but didn’t significantly rescue the expression of *bgs1*, *agn2* and *adg1* (Fig 7B; S5 and S6 Tables). The reduced expression of endo-1,3-alpha-glucosidases *agn1* and *agn2* in Nup211_1-655_ cells explained the phenotypes we observed, such as multi-septated and long cells (Fig 3B). In summary, these results suggest that Nup211 plays a role in transcriptional regulation and its N-terminal 655 amino acids are able to carry out part of its regulatory functions.

## Discussion

In this study, we determined that the N-terminal 655 amino acids of fission yeast essential nuclear pore protein Nup211 are sufficient for cell viability. We also revealed a new role for Nup211 in regulating gene expression.

Since *nup211* is an essential gene [8], we constructed a *nup211* shut off (*nup211-so*) strain in which the expression of *nup211* can be inhibited by thiamine to study its functions (Fig 1). As expected, down-regulating *nup211* expression significantly reduced cell viability (Fig 2). When *nup211* expression was shut off, we observed various cell cycle defects. Among them were different septation defects such as misplaced, thicker, or multiple septa (Fig 3). Ectopic expression of Nup211 rescued these phenotypes, indicating that the defects were specifically due to loss of Nup211 (Fig 4). Taken together, these results show that Nup211 plays roles in regulating cell division, including septation and cell separation. Using this *nup211-so* strain for the domain analysis, we found that Nup211_1-655_ was sufficient to rescue the lethal phenotype, suggesting that Nup211_1-655_ carries out the essential function(s) of Nup211 (Fig 4). Furthermore, we used the CRISPR/cas9 system and successfully generated a *nup211*_*1-655*_ strain. The truncation mutant cells grew well, confirming that the essential region of Nup211 is present in its first 655 amino acids (Fig 5A).

To our surprise, even though we were able to detect Nup211_1-655_ in the cell lysate by Western blot, the level was extremely low (Fig 5B). We were unable to detect Nup211_1-655_ by immunofluorescence microscopy. Both Nup211_1-863_ and Nup211_1-655_ are under the control of the endogenous *nup211* promoter in the CRISPR truncation strains, however, Nup211_1-655_ was detected at a much lower amount in cell lysate compared to Nup211_1-863_ (Fig 5B). Similar differences were observed in lysates obtained from *nup211-so* rescue conditions, where Nup211 fragments were expressed from the same vector (Fig 4B). Thus, we reason that the abundance of truncated Nup211 is more likely to be affected by protein stability. As shown in Fig 5B, transforming the Nup211_1-655_ expression plasmid pARS4 into the *nup211*_*1-655*_ strain, the protein level was significantly increased. We also observed degradation products when Nup211_1-655_ was overexpressed, indicating that there are several sensitive cleavage sites in this region. Using this Nup211_1-655_ overexpression strain, Nup211_1-655_ was detected at the nuclear periphery, showing a similar localization pattern as full-length Nup211 (Fig 5C). Our results showed that both Nup211_1-655_ and Nup211_1-863_ were localized at the NPCs, implying that these truncated Nup211 proteins play roles at the nuclear periphery.

Nuclear pore basket proteins have been shown to regulate chromatin organization and transcription in addition to nucleocytoplasmic transport [6]. Several orthologs of Nup211 have been implicated in transcriptional regulation [42, 48–50, 55]. To test whether Nup211 also regulates transcription, we performed RNA-Seq, followed by RT-qPCR. Our RNA-Seq analysis revealed that inhibiting *nup211* resulted in a significant change in the transcriptome. Among the affected genes, several are important for cytokinesis, which is consistent with the phenotypes we observed (see Results). The RNA-Seq results were confirmed by RT-qPCR analyses (Fig 7A). Furthermore, ectopically expressing the full-length protein restored the transcript levels of all the genes examined (Fig 7B). Interestingly, expressing Nup211_1-655_ in shut-off cells restored expression of most, but not all, of the genes analyzed (Fig 7B). This indicates that the deleted C-terminal region is important for Nup211’s function in regulating gene expression, even though it is not required for viability.

Nup211 may control gene expression at multiple levels. In addition to the role in regulating mRNA export [12, 13], here we report that Nup211 controls the mRNA levels of several genes involved in cytokinesis. Currently, the mechanism by which Nup211 regulates the transcriptome is not understood. Located at the nuclear basket, Nup211 may serve as anchors for positioning certain regions of chromatin near the NPCs. Recently, Iglesias *et al*. showed that Nup211 preferentially associates with heterochromatin, such as pericentromeric regions [46]. Its budding yeast orthologs, Mlp1 and Mlp2, are important for positioning genes at the nuclear pores for their transcriptional activation [33, 35, 51]. For example, Mlp1 interacts with components of the SAGA complex and activates GAL gene transcription [33]. The *Drosophila* ortholog Megator controls the transcription level of X chromosome genes and associates with transcriptionally active regions [48, 53, 56]. Moreover, the mammalian TPR is important for establishing heterochromatin boundaries around the NPCs [43, 44, 52]. Nup211 may also regulate transcription at the chromatin level. Our ongoing work to determine how Nup211 interacts with chromatin will shed light on the mechanisms by which it regulates chromatin organization and transcription.

## Conclusions

The essential yeast nucleoporin Nup211 regulates the expression of a set of genes involved in cytokinesis. Nup211_1-655_ is sufficient for viability and exhibits partial gene regulatory functions.

## Materials and methods

### Yeast strains, growth conditions and genetic manipulations

*Schizosaccharomyces pombe* strains used in this study are listed in (S1 Table). Strain *558* (*leu1-32 ura4-D18 ade6-M210 h+*) served as the wild-type control and was used to generate the remaining yeast strains. The *nup211-so* strain (*nup211*::*P81nmt1 ura4*^*+*^ *leu1-32 ura4-D18 ade6-M210 h+*) was engineered by replacing the endogenous *nup211* promoter with the *ura4*^*+*^ gene and thiamine-repressible promoter P81nmt. Additionally, two *nup211* truncation strains were created using the SpEdit method [47]. Primers for generating the guide RNAs and homologous recombination templates used to delete specific regions of the *nup211* ORF were designed using the CRISPR4P tool (S2 Table) [57]. Following the hybridization of sgRNA oligonucleotides, specific sgRNA sequences were integrated into the spCas9-containing pLSB-NAT plasmid using the Golden Gate Assembly kit (NEB #E1601S). Oligonucleotides for generating homologous recombination (HR) templates were annealed and amplified by PCR.

HR template DNA and *nup211*-specific pLSB-NAT plasmids were co-transformed into wild-type cells by electroporation. Cells were initially spread on EMM-NH_4_Cl plates and left to grow overnight at room temperature before being replica-plated to YES + NAT plates. After 4–7 days of growth at 30°C, candidate colonies were streaked onto non-selective YES plates and allowed to grow for an additional 2–3 days. Candidates were screened for the desired mutations by colony PCR using primers suggested by CRISPR4P. Strains were further validated by western blotting and DNA sequencing of deletion junctions.

For normal *nup211* expression, wild-type and *nup211-so* strains were grown in appropriately supplemented Edinburgh minimal media (EMM) lacking thiamine (https://dornsife.usc.edu/pombenet/media). To reduce *nup211* expression, *nup211-so* cells were grown in media containing 30uM (10 μg/mL) thiamine (EMM+T). Cells were grown for 24 hours, washed once with EMM+T, and inoculated in fresh EMM+T at OD_600_ = 0.05. The cells were then allowed to grow for an additional 24 hours before being used in downstream analyses. Plasmids used in rescue experiments were introduced into *nup211-so* cells using the lithium acetate method [58].

For growth assays involving *nup211* truncation strains, all cells were grown on pombe glutamate medium (PMG) plates supplemented with 225 mg/L adenine, leucine, uracil. For immunofluorescence microscopy, all strains were grown in appropriately supplemented liquid EMM media and harvested at an OD_600_ ≈ 0.3.

### Genomic DNA extraction

Genomic DNA was isolated using the phenol-chloroform method. Specifically, cells were harvested at mid-log phase and resuspended in DNA lysis buffer (100 mM Tris, pH 8.0; 100 mM NaCl; 1 mM EDTA, pH 8.0; 1% SDS). Acid washed beads were added to the suspensions, which were then vortexed for 5 min. Following centrifugation for 10 min at 10,000 rpm, the supernatant was mixed with phenol:chloroform (1:1) solution and centrifuged for 2 min. After two more extractions, the resulting aqueous layer was then mixed with 70% ethanol and centrifuged to precipitate DNA. The pellets were subsequently resuspended in 100 μL of 20 mg/mL RNaseA in 1X Tris-EDTA (pH 8.0).

### Southern blotting

Genomic DNA was digested with BglII overnight at 37°C and separated on a 0.8% agarose gel at 100V for 4 hours. The resulting DNA fragments were then transferred to a nitrocellulose Hybond-N+ membrane (Amersham). A probe was synthesized using primers designed to target the upstream *nup211* coding sequence (S2 Table) and radiolabeled with p32 using the Prime It II Random Primer Labeling Kit (Agilent Technologies). The membrane was incubated with pre-hybridization buffer (Sigma-Aldrich) and denatured salmon sperm DNA. Hybridization of the probe to the membrane was carried out using QuickHyb solution (Stratagene), followed by washing with either 2X SSC/0.1% SDS or 0.5X SSC/0.1% SDS. The membrane was then wrapped in saran wrap and placed on a PhosphorImager screen for viewing.

### Plasmids

Plasmids for exogenously expressing domains of Nup211 were constructed using the pLEV3 vector (gift from Dr. Charlie Hoffman, Boston College), which we modified through the addition of ApaI and NotI restriction sites to the multiple cloning site (MCS). Regions of the *nup211* open reading frame (ORF) were amplified from genomic DNA via PCR using forward and reverse primers containing ApaI and NotI restriction sites, respectively (S2 Table). PfuUltra II Fusion HS DNA Polymerase (Agilent Technologies) was used in all reactions. PCR products were digested with ApaI and NotI and cloned into the modified pLEV3 (mpLEV3) vector downstream of the *adh1* promoter. Junction regions were sequenced to confirm correct ligation.

### Antibodies

Primary antibodies used include mouse and rabbit anti-Nup211 (Immune Tech Corp), anti-β-actin (Abcam; ab8224), and mAb414 (Biolegend; Cat # 902902), a mouse monoclonal antibody that recognizes several nucleoporins. For Western blotting experiments, Pierce® goat anti-mouse poly-HRP (Thermo Scientific; Cat # 32230) was used as the secondary antibody. Alexa Fluor® 488 AffiniPure™ F(ab’)_2_ fragment goat anti-mouse IgG, Fcγ fragment specific (Jackson ImmunoResearch; 115-546-071) and Alexa Fluor® 647 AffiniPure™ F(ab’)_2_ fragment goat anti-rabbit IgG, Fc fragment specific (Jackson ImmunoResearch; 11-606-046) secondary antibodies were used in immunofluorescence experiments.

### Western blotting

Whole cell protein lysates were prepared from wild-type and *nup211-so* cells using the trichloroacetic acid (TCA) precipitation method [59]. Approximately 3 OD units of exponentially growing cells were subjected to two, 35-second rounds of disruption in a Thermo Savant FastPrep FP120 bead beater located in a cold room and set to 4.0 m/s. After each round, tubes were placed in an ice-water bath for 4 min to prevent protein degradation. Lysates were mixed with the appropriate volume of 5X SDS sample buffer, boiled for 5 min at 95°C, and cleared by centrifugation at 16,100 x g for 5 min at room temperature.

For Nup211 truncation strains, whole cell protein lysates were prepared using a modified version of the protocol outlined in [58]. Approximately 20 OD units of exponentially growing cells were harvested and washed once ice-cold lysis buffer (50 mM Tris-HCl pH 7.5, 150 mM NaCl, 10 mM MgCl_2_, 1 mM EDTA, 10% Glycerol, 1 mM DTT, 1 mM PMSF and 1 × protease inhibitor cocktail (Roche)). Cell pellets were resuspended in 200 μl of ice-cold lysis buffer and mixed with 500 μL of ice-cold beads in 2 mL screw-cap tubes. Cells were subjected to two, 35-second rounds of disruption in a Thermo Savant FastPrep FP120 bead beater as described above. After, screw-cap tubes were punctured at the bottom using a 21 G needle and lysates were collected in 1.5 mL microcentrifuge tubes by centrifugation (2 min, 500 x g, 4°C). Beads were then washed by adding an additional 200 μL of lysis buffer to the screw-cap tube repeating centrifugation. Lysates were cleared by spinning at 16,100 x g for 5 min at 4°C. The cleared supernatant was transferred to a fresh 1.5 mL microcentrifuge tube and mixed with the appropriate volume of 5X SDS sample buffer before boiling at 95°C for 10 min.

Protein samples were separated on 4–16% SDS-PAGE gels and transferred onto a nitrocellulose membrane (Bio-Rad) in a cold room for 90 min at 75 V. The nitrocellulose membrane was blocked in 5% non-fat milk in 1X PBST (0.05% Tween-20) for 1 h. Membrane strips were incubated with primary antibodies overnight at 4°C. Next, strips were rinsed three times (5 min each) in 1X PBST (0.05% Tween-20) and probed for 40 min with secondary antibodies conjugated to polyHRP in 5% non-fat milk. After three 5-min washes, secondary antibody binding was detected using Amersham^™^ ECL^™^ Western Blotting Detection Reagents (Cytiva).

### Microscopy

For live cell imaging, cells were mounted onto agarose pads prepared as previously described [60]. Briefly, cells were picked from EMM plates containing or lacking thiamine, resuspended in 1 mL ddH_2_O, and centrifuged for 15 sec at 13,000 rpm. Alternatively, cells growing in liquid EMM (± T) were harvested at mid log phase, washed once with ddH_2_O, and centrifuged for 15 sec at 13,000 rpm. Small amounts of the resulting pellets were mounted onto 2% agarose pads to prevent cell migration during imaging.

Nuclei and cell wall staining was performed as described previously, with some modifications [61]. First, 2% formaldehyde was added directly to the culture medium to fix cells. After several washes with cold PBS, cells were resuspended in PBS containing 1 μg/mL DAPI (Sigma-Aldrich) and 0.5 mg/mL Aniline Blue (Fisher Chemical). Following a 15-min incubation period at room temperature, stained cells were centrifuged for 2 min at 3,000 rpm and the resulting pellets were mounted onto 2% agarose pads. Microscopy was performed using the Nikon Eclipse Ti Fluorescence Microscope equipped with an Andor iXon EMCCD camera and a DG5 wavelength switcher. Images were further analyzed using FIJI (NIH). Figures presenting microscopy data were produced using the Adobe Creative Suite (Adobe Photoshop and Illustrator) and the ScientiFig plugin for FIJI.

For immunofluorescence staining, cells were cultured to mid-log phase (OD_600_ ≈ 0.3–0.6) and harvested by centrifugation at 3000 x g for 3 min. The resulting cell pellets were resuspended in 1 mL of PBS, transferred to microcentrifuge tubes, and subjected to two additional washes with PBS. To digest the cell wall material, cells were resuspended in Spheroplasting buffer (1X PBS, 1.2M sorbitol, 500 U lyticase) and incubated in a 30°C shaker for 30–60 min. After three PBS washes, cells were fixed in 1 mL of fixation solution (0.1M potassium phosphate, pH 6.5, 10% methanol v/v, 3.7% w/v formaldehyde) for 25 min on a rotator at room temperature. Cells were then permeabilized in 1 mL of permeabilization solution (1X PBS, 0.1% Triton-X-100, 1% BSA) for 25 min. After carefully washing away the permeabilization buffer and resuspending in 1 mL of PBS, 100 μL of permeabilized cells were spread on poly-L-lysine coated microscope slides and allowed to sit for 1 hour. Cells were heat-fixed on a heating block warmed to 70°C for 60–90 sec. and blocked in a humid chamber for 30–60 min at room temperature by adding 100 μL of blocking solution (1X PBS, 3%BSA, 0.1M lysine HCl) to each cell spot. After removing the blocking solution, cells were incubated in 100 μL of primary antibody (diluted in blocking solution) in the humid chamber overnight at room temperature. Following three washes with 1 mL PBS, 100 μL of diluted secondary antibody solution was added and slides were incubated for one hour in a darkened humid chamber at room temperature. The secondary antibody solution was then washed away, and slides were air-dried in the dark before mounting solution was added to the fixed cell spots and covered with a final coverslip. Slides were viewed using a Leica TCS SP2 Laser Scanning Spectral Confocal Microscope.

### RNA extraction

For cell wall digestion, 5x10^7^ cells were incubated with 250 μg of lyticase (Sigma-Aldrich) for 1–2 hours at 30°C. After spheroplasting was complete, total RNA were extracted from cells using the RNeasy Kit (Qiagen) following the manufacturer’s instructions. RNA concentration and quality were measured using a NanoDrop ND-1000 Spectrophotometer.

### RT-qPCR

Triplicate RNA samples were prepared for each experimental condition. The QuantiTect Reverse Transcription Kit (Qiagen) was used to synthesize cDNA from total RNA samples. Subsequent qPCR was performed using the QuantiTect SYBR Green PCR Kit (Qiagen) and the ViiA7 qPCR machine (Applied Biosystems) following the manufacturers’ instructions. Changes in relative transcript levels were calculated using the ΔΔCq method, with *act1* serving as the endogenous control. Statistical analysis was performed in GraphPad Prism. To assess the statistical significance of differential expression observed upon shutting off *nup211* expression, multiple Welch’s t-tests were performed using the Holm-Šídák method (alpha = 0.05) to correct for multiple comparisons. For rescue experiments, Fisher’s Least Significant Difference (LSD) test was performed following two-way ANOVA to determine if the abundance of individual transcripts were significantly affected by expressing additional Nup211 in *nup211* shut-off cells.

## Supporting information

S1 Table Fission yeast strains and plasmids used in this study. (DOCX)

S2 Table Primers used in this study. (DOCX)

S3 Table Summary of RNA-seq results. RNA-sequencing data was analyzed in R using the DESeq2 program. The table shows the number of differentially expressed transcripts at two different fold change cutoff values that satisfy the p-value threshold (10^−6^) used to generate the volcano plot shown in Fig 6.
(DOCX)

S4 Table Log_2_ fold-change and p-values for *nup211-*so RT-qPCR experiments. (DOCX)

S5 Table Log_2_ relative mRNA expression data for *nup211-so* rescue RT-qPCR experiments. (DOCX)

S6 Table p-values for *nup211-so* rescue RT-qPCR experiments by ANOVA. (DOCX)

S1 Raw image Original images for Western blots. Raw images of Western blot results presented in Figs 2A, 4B, and 5B.
(PDF)
