# Supplementary material for: Fission yeast essential nuclear pore protein Nup211 regulates the expression of genes involved in cytokinesis
Source: PLoS One. 2024 Dec 12;19(12):e0312095. doi: 10.1371/journal.pone.0312095 (PMC11637317; doi:10.1371/journal.pone.0312095)
Supplement: S1 Table — (DOCX) [file pone.0312095.s001.docx]

**S1 Table. Fission yeast strains and plasmids used in this study.**

| **Fission Yeast Strains** | | | | |
| --- | --- | --- | --- | --- |
| **Name** | | **Genotype** | | **Source** |
| 558 (*nup211-wt*) | | *leu1-32 ura4-D18 ade6-M210 h+* | | Nurse Lab |
| *nup211-so* | | *nup211::nmt1 ura4^+^ -leu1-32 ura4-D18 ade6-M210 h+* | | This study |
| *nup211_1-863_* | | *nup211_1-863_* *leu1-32 ura4-D18 ade6-M210 h+* | | This study |
| *nup211_1-655_* | | *nup211_1-655_* *leu1-32 ura4-D18 ade6-M210 h+* | | This study |
| **Plasmids** | | | | |
| **Name** | **Selection Marker** | **Restriction**  **Sites** | **Gene Product / Insert** | |
| mpLEV3 | *LEU2* | N/A | N/A | |
| pARS1 | *LEU2* | NotI/ApaI | Nup211_1-1837_ | |
| pARS2 | *LEU2* | NotI/ApaI | Nup211_1-1033_ | |
| pARS3 | *LEU2* | NotI/ApaI | Nup211_1-863_ | |
| pARS4 | *LEU2* | NotI/ApaI | Nup211_1-655_ | |
| pARS5 | *LEU2* | NotI/ApaI | Nup211_1-412_ | |
| pARS6 | *LEU2* | NotI/ApaI | Nup211_1033-1837_ | |
| pLSB | *natMX6* | BsaI | N/A | |
| pDK1998 | *natMX6* | BsaI | CAAGTCGAATAAGCGAACGC | |
| pDK2922 | *natMX6* | BsaI | CCAAGGTTTTTTCTGAAGCG | |
