# Supplementary material for: Fission yeast essential nuclear pore protein Nup211 regulates the expression of genes involved in cytokinesis
Source: PLoS One. 2024 Dec 12;19(12):e0312095. doi: 10.1371/journal.pone.0312095 (PMC11637317; doi:10.1371/journal.pone.0312095)
Supplement: S2 Table — (DOCX) [file pone.0312095.s002.docx]

**S2 Table. Primers used in this study.**

| **Primers for CRISPR Editing** | |
| --- | --- |
| **Name** | **Sequence (5′-3′)** |
| sg1998F | CTAGAGGTCTCGGACTCAAGTCGAATAAGCGAACGCGTTTCGAGACCCTTCC |
| sg1998R | GGAAGGGTCTCGAAACGCGTTCGCTTATTCGACTTGAGTCCGAGACCTCTAG |
| sg2922F | CTAGAGGTCTCGGACTCCAAGGTTTTTTCTGAAGCGGTTTCGAGACCCTTCC |
| sg2922R | GGAAGGGTCTCGAAACCGCTTCAGAAAAAACCTTGGAGTCCGAGACCTCTAG |
| HRdel656F | AACGAGACTTTTTTAGATCACTAGTTCAAGAGAATGAAAAACTTTTAGAT  ATGGCGCCTGCAACTCCTAATTCCAAATTGTAGATGTTTTCATATGGTTC |
| HRdel656R | ACTCATGTCATTATTATAAATCATGTTAACTAAATATGAATAGTCCTAAG  AGTGATTTATGAACCATATGAAAACATCTACAATTTGGAATTAGGAGTTG |
| HRdel864F | TGAATAGTCGAATACTTGAACTTTCTAATGATCTTCGAGTTGCTAATTCA  AAGCTATCTGAGTGTTCCGATGATGTTCGTTAGATGTTTTCATATGGTTC |
| HRdel864R | ACTCATGTCATTATTATAAATCATGTTAACTAAATATGAATAGTCCTAAG  AGTGATTTATGAACCATATGAAAACATCTAACGAACATCATCGGAACACT |
| Ck5primeF | CTACCTCGTCGCTACACACC |
| Ck656Fw | AATGTTGGAGGAGGTGAGCG |
| Ck864Fw | GAAACTTTCGTCCCTGCAGC |
| Ck1837Rv | ACTTCTGCAAATAGTCGTGGT |
| **Primers for quantitative real-time PCR** | |
| **Primer name** | **Sequence (5′-3′)** |
| Atf1-**F** | GCCATCTGTTTACGGCGATAC |
| Atf1-**R** | CCATTCTCGGCATTTTGTCCG |
| Bgs1-**F** | ACGGGTATGGCGGAACAAAT |
| Bgs1-**R** | TGAAATCCAGCGTGGGCATA |
| Knh1-**F** | GCGTCTTTGATGGCTCTTGG |
| Knh1-**R** | CGCATTGTACCTGTAGGCCA |
| Mbx1-**F** | TCATCTCCTCGCCGTTCTATAC |
| Mbx1-**R** | GACCACCACTTTAGCATCAC |
| Ace2-**F** | TCTGAGGATTCACGAAAATGGC |
| Ace2-**R** | TCATTAGTGCTGTCTGCGATCT |
| Pom1-**F** | GGGTTTGACGACGAAAGAGGA |
| Pom1-**R** | AGAACCTGTCCAAAACTCCCC |
| Pxl1-**F** | ACTAATGGCTCCCCGCTAAAC |
| Pxl1-**R** | ACTTGGTTGCGGTAATGGTGT |
| Agn1-**F** | CCGGTGGTGATTATATGGAAGAT |
| Agn1-**R** | CCACTGAATGTTTGTGTTGTAGAG |
| Agn2-**F** | GCACATTTCTTGGGCAGGAT |
| Agn2-**R** | GATACCAGCAGAAGCCAACG |
| Adg1-**F** | TGGACACTCATAACCCTCTTTC |
| Adg1-**R** | GAGCTAACACGGTCTCCTTTAT |
| Act1-**F** | ACGTCGCTTTGGACTTTGA |
| Act1-**R** | CGCTCGTTTCCGATAGTGATAA |
| **Primers for generating and verifying *nup211-so*** | |
| **Primer name** | **Sequence (5′-3′)** |
| Nmt-**F** | AGACAGAATAAGTCATCAGCGGTTGTTTC |
| Nup211-**R** (nt384-412) | GAGCAGTTTGCTTCAAATTTTGATCCTG |
| Ura+Nmt Chimeric | CATATCAGCAAAGACTTTCTCAGCATTAA  AGACAGAATAAGTCATCAGCGGTTGTTTC |
| Primer 1 | CCTCGTCGCTACACACCC |
| Primer 2 | GCTGGCTATTACAGATTGATAATTGAG |
| Primer 3 | TTAATGCTGAGAAAGTCTTTGCTGATATG |
| Primer 4 | GGAATTAGGAGTTGCAGGCG |
| Southern Probe-**F** | GATGCGTCGTCTAGGATCCAAATTTTG |
| Southern Probe-**R** | CGCTTGGTGATTCAATGTTGGAC |
