# Supplementary material for: Fission yeast essential nuclear pore protein Nup211 regulates the expression of genes involved in cytokinesis
Source: PLoS One. 2024 Dec 12;19(12):e0312095. doi: 10.1371/journal.pone.0312095 (PMC11637317; doi:10.1371/journal.pone.0312095)
Supplement: S3 Table — RNA-sequencing data was analyzed in R using the DESeq2 program. The table shows the number of differentially expressed transcripts at two different fold change cutoff values that satisfy the p-value threshold (10−6) used to generate the volcano plot shown in Fig 6. (DOCX) [file pone.0312095.s003.docx]

**S3 Table: Summary of RNA-seq results.**

|  | Differentially Expressed Transcripts | | |
| --- | --- | --- | --- |
| Log_2_FC Cutoff | Upregulated | Downregulated | Total |
| Two-fold | 629 | 394 | 1023 |
| Four-fold | 193 | 108 | 301 |

RNA-sequencing data was analyzed in R using the DESeq2 program. The table shows the number of differentially expressed transcripts at two different log_2_(fold change) cutoff values that satisfy the p-value threshold (10^-6^) used to generate the volcano plot shown in Figure 6.
