# Supplementary material for: Fission yeast essential nuclear pore protein Nup211 regulates the expression of genes involved in cytokinesis
Source: PLoS One. 2024 Dec 12;19(12):e0312095. doi: 10.1371/journal.pone.0312095 (PMC11637317; doi:10.1371/journal.pone.0312095)
Supplement: S4 Table — (DOCX) [file pone.0312095.s004.docx]

**S4 Table. Log_2_ fold-change and p-values for *nup211-*so RT-qPCR experiments.**

|  | *nup211-so* + Thiamine | | | | | | |
| --- | --- | --- | --- | --- | --- | --- | --- |
|  | log2 Relative mRNA expression | | | | 95% Confidence Interval | |  |
| gene | Replicate 1 | Replicate 2 | Replicate 3 | Mean | Upper Limit | Lower Limit | P-value |
| atf1 | 2.08 | 1.86 | 2.13 | 2.02 | 2.39 | 1.66 | 0.00395 |
| mbx1 | 3.50 | 3.41 | 3.60 | 3.50 | 3.74 | 3.27 | 0.00074 |
| pom1 | 1.04 | 0.89 | 1.12 | 1.02 | 1.31 | 0.72 | 0.00395 |
| knh1 | 1.98 | 2.01 | 2.16 | 2.05 | 2.29 | 1.81 | 0.00014 |
| pxl1 | 1.74 | 1.76 | 1.81 | 1.77 | 1.85 | 1.68 | 0.01448 |
| bgs1 | 1.59 | 1.52 | 1.81 | 1.64 | 2.02 | 1.26 | 0.00159 |
| agn1 | -0.95 | -0.76 | -0.97 | -0.90 | -0.61 | -1.19 | 0.02355 |
| agn2 | -1.05 | -1.18 | -1.03 | -1.09 | -0.89 | -1.28 | 0.00842 |
| adg1 | -2.43 | -2.15 | -2.56 | -2.38 | -1.86 | -2.90 | 0.00672 |
| ace2 | -1.17 | -0.16 | -0.48 | -0.60 | 0.68 | -1.89 | 0.23498 |
