# Supplementary material for: Fission yeast essential nuclear pore protein Nup211 regulates the expression of genes involved in cytokinesis
Source: PLoS One. 2024 Dec 12;19(12):e0312095. doi: 10.1371/journal.pone.0312095 (PMC11637317; doi:10.1371/journal.pone.0312095)
Supplement: S5 Table — (DOCX) [file pone.0312095.s005.docx]

**S5 Table. Log_2_ relative mRNA expression data for *nup211-so* rescue RT-qPCR experiments.**

|  |  | log2 Relative mRNA expression | | | | 95% Confidence Interval | |
| --- | --- | --- | --- | --- | --- | --- | --- |
| gene | *nup211-so +* | Replicate 1 | Replicate 2 | Replicate 3 | Mean | Upper Limit | Lower Limit |
| atf1 | empty vector | 2.75 | 2.21 | 2.72 | 2.56 | 3.32 | 1.80 |
| atf1 | Nup211_full-length_ | 1.07 | 0.81 | 0.84 | 0.91 | 1.27 | 0.55 |
| atf1 | Nup211_1-655_ | 0.64 | 0.64 | 0.55 | 0.61 | 0.73 | 0.49 |
| mbx1 | empty vector | 4.16 | 4.65 | 4.86 | 4.56 | 5.44 | 3.67 |
| mbx1 | Nup211_full-length_ | 0.78 | 0.37 | 0.56 | 0.57 | 1.07 | 0.07 |
| mbx1 | Nup211_1-656_ | 0.76 | 0.76 | 0.55 | 0.69 | 0.99 | 0.39 |
| pom1 | empty vector | 1.56 | 2.07 | 1.72 | 1.78 | 2.43 | 1.14 |
| pom1 | Nup211_full-length_ | 0.51 | 0.17 | 0.19 | 0.29 | 0.77 | -0.18 |
| pom1 | Nup211_1-657_ | 0.23 | 0.23 | 0.00 | 0.15 | 0.47 | -0.17 |
| knh1 | empty vector | 2.72 | 2.23 | 2.63 | 2.53 | 3.18 | 1.88 |
| knh1 | Nup211_full-length_ | 0.67 | -0.41 | 0.46 | 0.24 | 1.66 | -1.19 |
| knh1 | Nup211_1-658_ | -0.62 | -0.62 | 0.32 | -0.31 | 1.04 | -1.65 |
| pxl1 | empty vector | 2.55 | 2.56 | 2.88 | 2.66 | 3.13 | 2.20 |
| pxl1 | Nup211_full-length_ | 0.87 | 0.17 | 0.29 | 0.44 | 1.38 | -0.50 |
| pxl1 | Nup211_1-658_ | 0.24 | 0.24 | 0.15 | 0.21 | 0.33 | 0.09 |
| bgs1 | empty vector | 2.52 | 3.50 | 2.77 | 2.93 | 4.20 | 1.66 |
| bgs1 | Nup211_full-length_ | 0.82 | 2.23 | 0.21 | 1.09 | 3.66 | -1.49 |
| bgs1 | Nup211_1-658_ | 2.21 | 2.21 | 0.04 | 1.49 | 4.59 | -1.62 |
| agn1 | empty vector | -0.95 | -1.18 | -1.13 | -1.08 | -0.78 | -1.39 |
| agn1 | Nup211_full-length_ | -0.23 | -0.57 | -0.34 | -0.38 | 0.05 | -0.81 |
| agn1 | Nup211_1-658_ | -0.72 | -0.86 | -0.79 | -0.79 | -0.61 | -0.96 |
| agn2 | empty vector | -0.85 | -0.99 | -0.81 | -0.88 | -0.65 | -1.12 |
| agn2 | Nup211_full-length_ | -0.09 | -0.08 | 0.00 | -0.06 | 0.07 | -0.19 |
| agn2 | Nup211_1-658_ | -1.33 | -1.45 | -1.06 | -1.28 | -0.79 | -1.78 |
| adg1 | empty vector | -2.15 | -2.00 | -1.74 | -1.97 | -1.45 | -2.48 |
| adg1 | Nup211_full-length_ | 0.28 | -0.04 | 0.16 | 0.13 | 0.54 | -0.27 |
| adg1 | Nup211_1-658_ | -1.23 | -1.60 | -1.49 | -1.44 | -0.97 | -1.91 |
