# Supplementary material for: Fission yeast essential nuclear pore protein Nup211 regulates the expression of genes involved in cytokinesis
Source: PLoS One. 2024 Dec 12;19(12):e0312095. doi: 10.1371/journal.pone.0312095 (PMC11637317; doi:10.1371/journal.pone.0312095)
Supplement: S6 Table — (DOCX) [file pone.0312095.s006.docx]

**S6 Table: p-values for *nup211-so* rescue RT-qPCR experiments by ANOVA.**

|  | P-value | |
| --- | --- | --- |
| gene | empty vector vs. Nup211_full-length_ | empty vector vs. Nup211_1-655_ |
| atf1 | 0.0072 | 0.0095 |
| mbx1 | 0.0056 | 0.0045 |
| pom1 | 0.026 | 0.0086 |
| knh1 | 0.0061 | 0.0109 |
| pxl1 | 0.0155 | 0.0031 |
| bgs1 | 0.0395 | 0.1754 |
| agn1 | 0.005 | 0.0136 |
| agn2 | 0.0028 | 0.0328 |
| adg1 | 0.0063 | 0.123 |
