# Supplementary figures and images for: Fission yeast essential nuclear pore protein Nup211 regulates the expression of genes involved in cytokinesis
Source: PLoS One. 2024 Dec 12;19(12):e0312095. doi: 10.1371/journal.pone.0312095 (PMC11637317; doi:10.1371/journal.pone.0312095)

Fig 2A

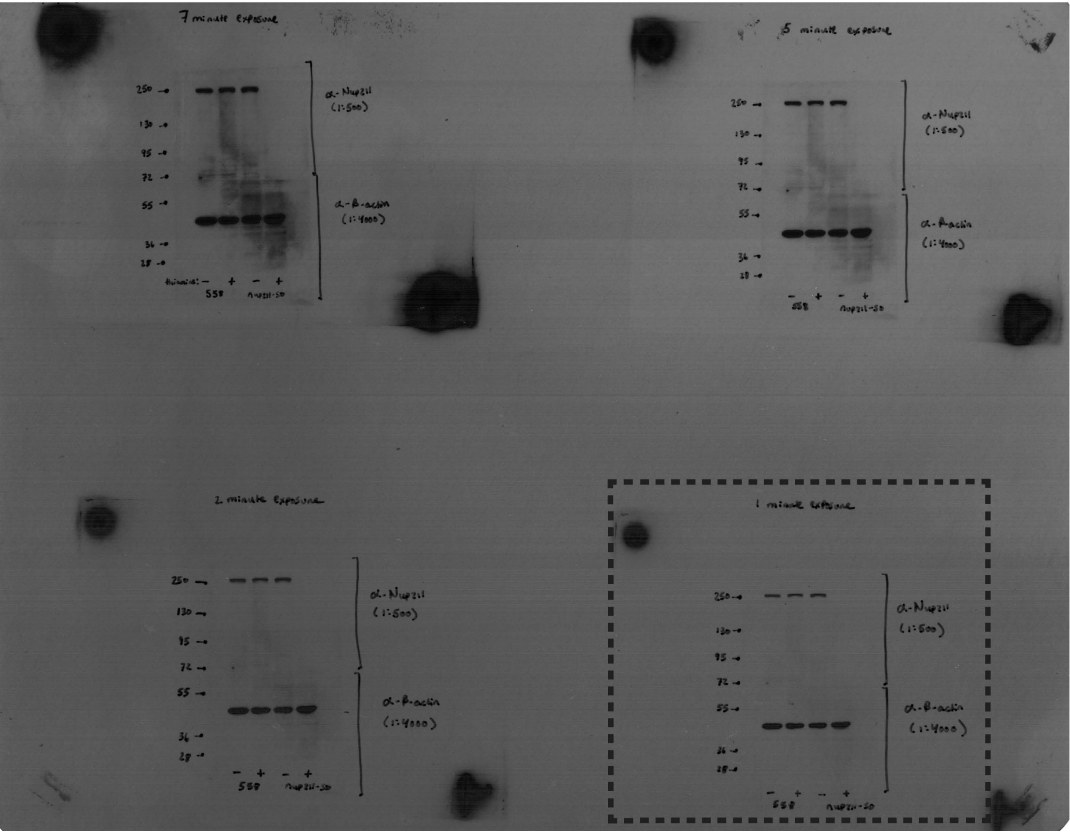

Fig 4B

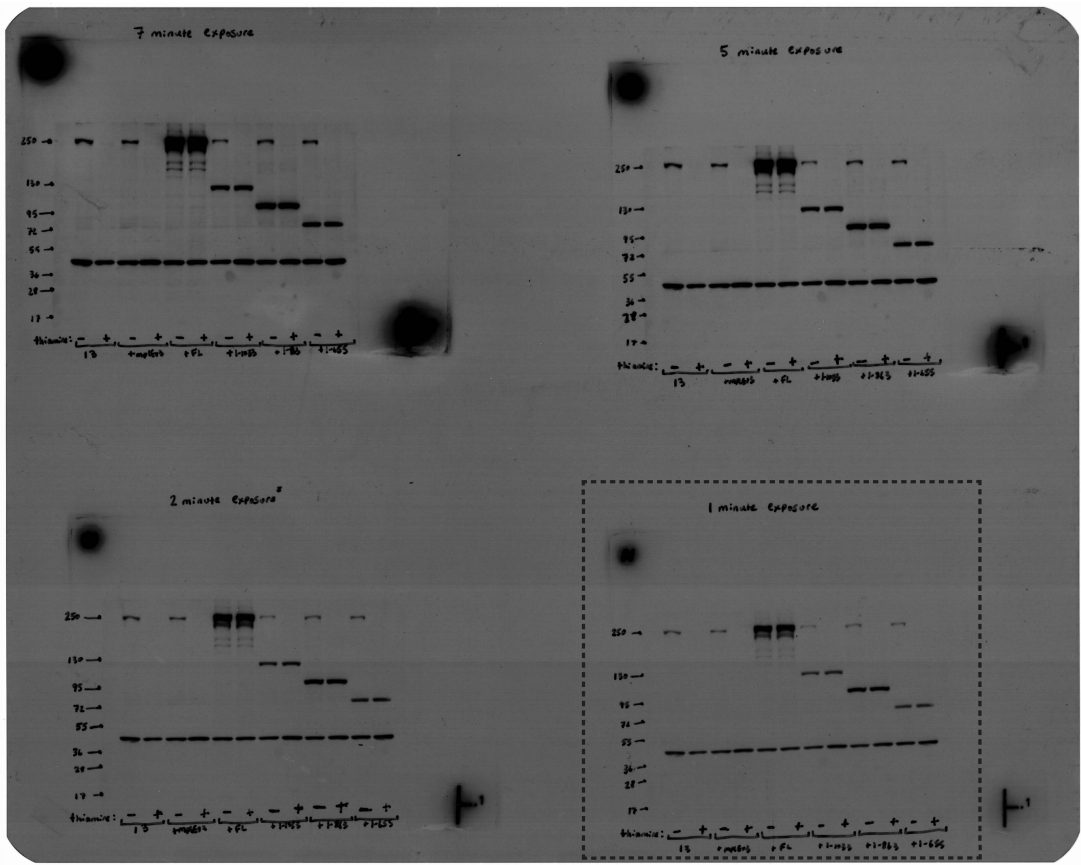

Fig 5B

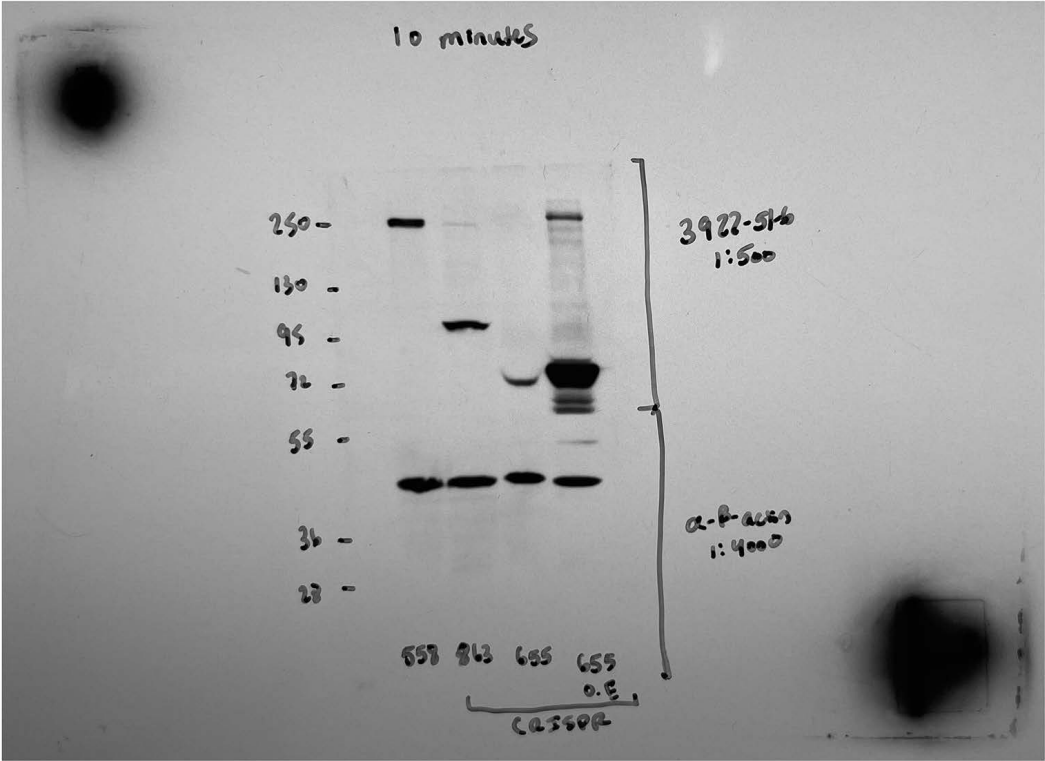

Supplement: S1 Raw image — Raw images of Western blot results presented in Figs 2A, 4B, and 5B. (PDF) [file pone.0312095.s007.pdf]
